# Resolutions of Respect to Dr. Otis Avery

**Published:** 1904-06

**Authors:** 


### Obituary.

### RESOLUTIONS OF RESPECT TO DR. OTIS AVERY.

THE Lackawanna-Luzerne Dental Society, at a regular meeting, held March 15, 1904, adopted the following resolutions upon the death of Dr. Otis Avery, an honored member of the Dental profession for more than seventy years, and who passed away in his ninety-sixth year.

WHEREAS, It has pleased the Divine Ruler to remove from this life Dr. Otis Avery, who passed to the great beyond February 22, 1904; and

WHEREAS, The dental profession recognizes the benefits received through his having lived, and by his life given us an example of a true, courteous, professional gentleman; therefore be it

*Resolved,* That in the death of Dr. Avery our profession has lost a man of sterling worth, whose progress in the profession was a source of pride to his colleagues. And for whose example we return thanks to the Divine Ruler; also,

*Resolved,* That we condole with his bereaved family, and a copy of the resolutions be sent to his widow and to the dental journals, and also be inscribed on our official records.

C. L. BECK,
W. A. SPENCER,
E. J. DONNEGAN,
*Committee.*

